# Bibliographical

**Published:** 1859-01

**Authors:** 


### BIBLIOGRAPHICAL.

### *Watson's Practice of Physic.*

We find upon our table, after long neglect, a number of books, pamphlets, &c., the most prominent perhaps, of which is our old friend, "Lectures on the Principles and Practice of Physic, delivered at King's College, London, by Thomas Watson, M. D.," in the shape of a new American, from the last revised English edition.

In this edition, the Lectures of Dr. Watson, have undergone a thorough revision, and whatever of value, recent research has added to our stock of knowledge, in the various departments of medical science, has been carefully incorporated in them. The Lectures on Fever especially have been greatly enlarged and improved; the positive distinctions that have been insisted upon by eminent Pathologists between typhus and typhoid fevers, are recognised as being founded in truth.

The extent of the additions to the work, is shown by the fact, that, notwithstanding a very considerable enlargement in the size of the page, the work has been increased by about two hundred pages.

To make the work more interesting to the American Physician, and to remedy some deficiencies which existed in the English Edition, Dr. Condie, the editor, has directed particular attention.

We can most warmly endorse the statement of the editor, that, "For comprehensiveness of matter, accuracy of detail, candor in the decision of those questions upon which a difference of opinion exists, among physicians, and perspicuity and felicity in the manner of presenting the several subjects embraced in them, they stand unrivalled."

In conclusion, we have to say, that it is one of the few works upon the Practice of Medicine, that we would be willing to undertake to read.

The work is issued from the press of Blanchard & Lea, Phila., and received at this office through Messrs. J. J. Richards & Co., of Atlanta, from whom it can be obtained.

### *Wilson's Human Anatomy.*

We are also in possesion of a system of Human Anatomy, general and special, by Erasmus Wilson, F. R. S., author of the Dissector's Manual, Treatise on Diseases of the Skin, &c., &c.—A new and improved American from an enlarged London Edition, and Edited by Wm. H. Gobrecht, M. D., Professor of Anatomy, in the Philadelphia College of Medicine.

Considering it a work of supererogation to say anything in praise of this standard work on Anatomy, we merely announce the new edition. It is issued by Blanchard & Lea, publishers, Phila., and reaches us through the hands of J. J. Richards & Co., of this city.

### *West on the Diseases of Women.*

We also find West on the Diseases of Women—a Second Part of the admirable Lectures published in 1856, by the same author, and which we have favorably noticed. Published by Blanchard & Lea, and sold by J. J. Richards & Co., in Atlanta, Ga.

### *Tyler Smith's Lectures on Obstetrics.*

We find ourselves also in receipt of "The Modern Practice of Midwifery," a course of Lectures on Obstetrics, delivered at St. Mary's Hospital, London, by Wm. Tyler Smith, M. D., &c. &c., with an introductory lecture on the History of the Art of Midwifery and copious Annotations, by Augustus K. Gardner, A. M., M. D., &c., of New York. This work is a collection (as the basis) of the Lectures which appeared in the London Lancet, and makes, we are confident, an admirable book on Obstetrics. The name of the author above, is sufficient to guarantee its merit, and we promise ourselves much pleasure and profit in its careful examination at some future period.

The work is published by Robt. W. DeWitt, New York City, and received through Messrs. J. J. Richards & Co., of this city, where will at all times be found a fine assortment of the best medical works, for sale, on favorable terms.

